# Supplementary material for: Shifts of Leaf Litter-Induced Plant-Soil Feedback from Negative to Positive Driven by Ectomycorrhizal Symbiosis between Quercus ilex and Pisolithus arrhizus
Source: Microorganisms. 2023 May 25;11(6):1394. doi: 10.3390/microorganisms11061394 (PMC10300854; doi:10.3390/microorganisms11061394)
Supplement: Supplementary file 1 [file microorganisms-11-01394-s001.zip › microorganisms-2404137-supplementary.pdf]

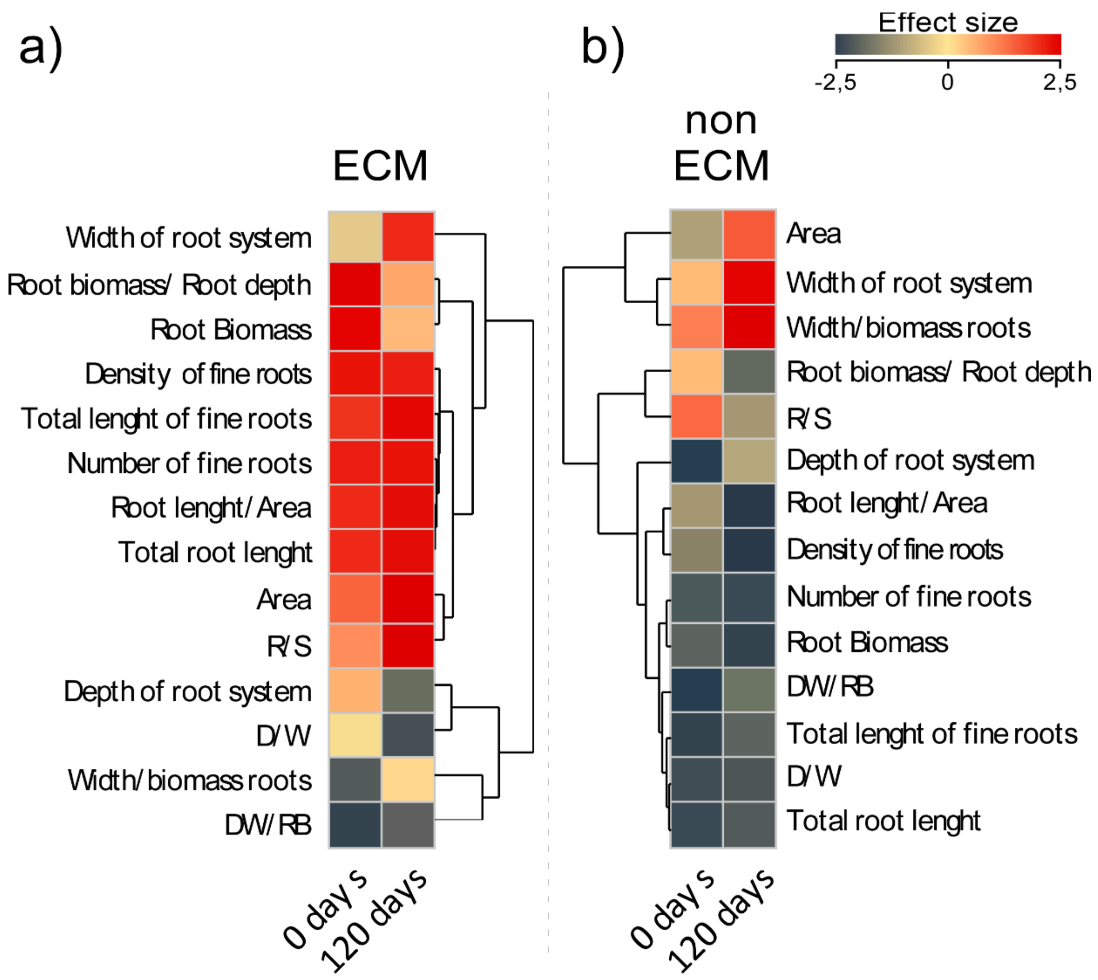

Figure S1. Heatmaps of effect size from *Q. ilex* seedlings biometric variables. Each of the variable were rescaled according to their effect respect no litter treatment in both ECM a) and non-ECM b) conditions for soils enriched with fresh (0d), aged (120 d) litters. In heatplot variables were ordered according to Euclidean distance hierarchical clustering.

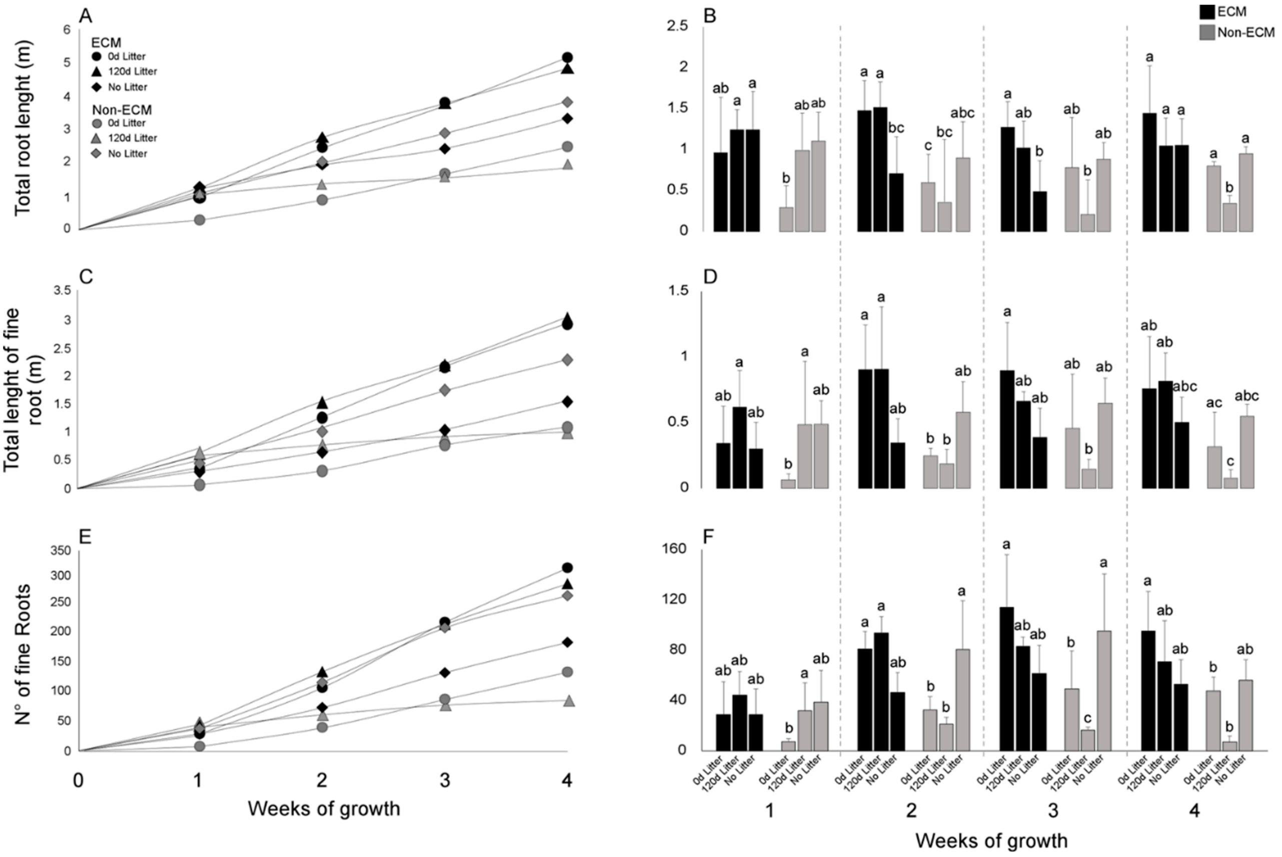

Figure S2 Cumulative root length of *Q. ilex* seedlings (a), Total length of fine roots (c), and Number of fine roots (e). the gain of total root length (b), Total length of fine roots (d), and Number of fine roots (f) measured every 15 days in ectomycorrhizal and non-ectomycorrhizal seedlings of *Q. ilex* with different litter regimes. Letters indicate significant variations assigned through Duncan post hoc test (p-value < 0,05).

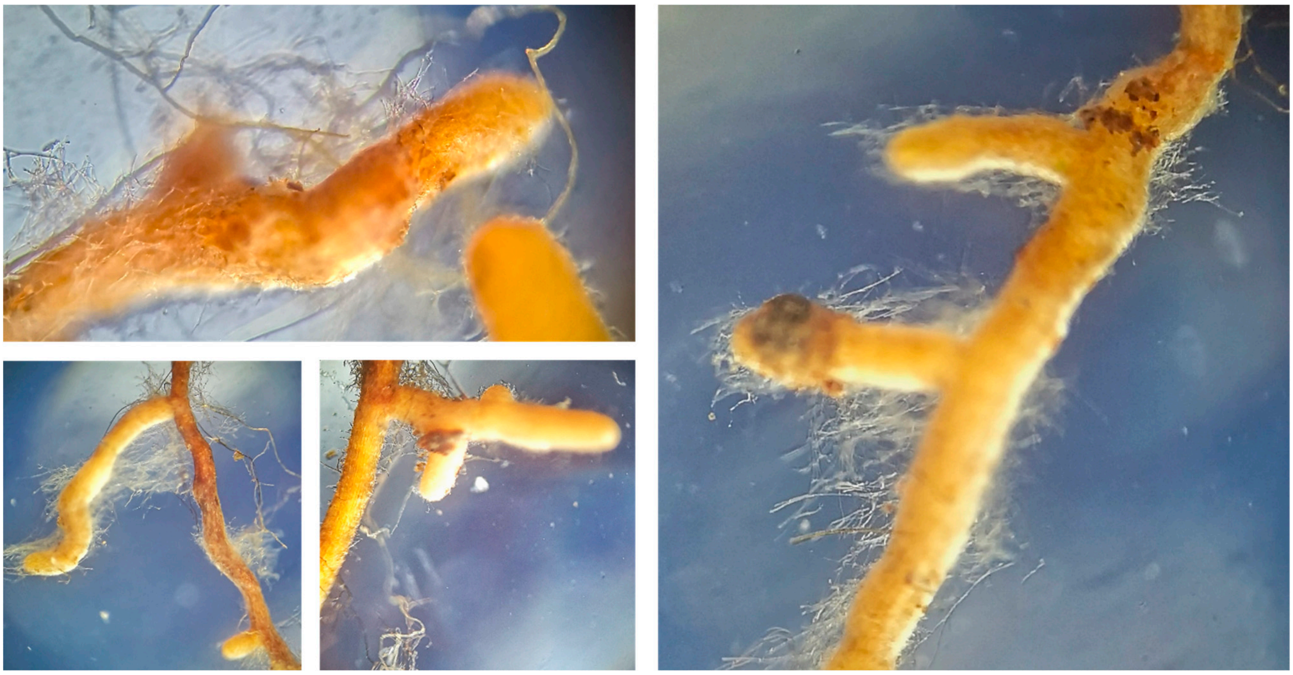

Figure S3 appearance of root tips of *Quercus ilex* after inoculation of *Pisolithus arrhizus* under dissection microscope at 4x. inoculated Seedling in a range between 15 and 30 % of root tips ECM colonization were included in the experiment and compared with non-inoculated seedlings. Seedlings below or exceeding the colonization threshold were not used in the experiment.

Table S1. GLM testing significant variations for all the parameters measured and estimated for *Q. ilex* seedlings growing in presence of ectomycorrhizal symbiosis of *P. tinctorius*, and litter age. Significant values in bold with *p-values* < 0,05

Parameters	Abbreviation	Plant portion	Data type	Factorial ANOVA		
				Litter age	ECM status	Litter age*ECM status
Width of the root system (cm)	WRS	Root	Measured	***0.044***	***0.006***	*0.584*
Depth of root system (cm)	DRS			***0.023***	*0.189*	***0.015***
Area of the root system (cm^2)	ARS			*0.124*	***0.003***	*0.340*
Total length of fine roots (cm)	TLFR			*0.825*	***0.031***	***0.023***
Number of fine roots	NFR			*0.763*	***0.023***	***0.011***
Total root length (cm)	TRL			*0.923*	***0.009***	*0.070*
Root Biomass (g)	RB			*0.392*	*0.065*	*0.070*
Shoot Biomass (g)	SB	Shoot	Measured	*0.247*	*0.834*	*0.071*
Stem Biomass (g)	StB			*0.380*	*0.872*	*0.136*
Shoot Lenght (cm)	SL			*0.263*	*0.199*	*0.145*
Number of leaves	NL			*0.492*	*0.562*	*0.246*
Leaves biomass (g)	LB			*0.178*	*0.812*	*0.056*
Total Biomass (gr)	TB	Total	Measured	*0.271*	*0.460*	*0.054*
Root length/Area	TLFR/ARS	Root	Estimated	*0.259*	*0.747*	*0.062*
Root biomass/ Root depth	RB/DRS			*0.065*	*0.161*	*0.291*
Root Width/ Root biomass	WRS/RB			***0.027***	*0.966*	***0.048***
Density N°root/area	NFR/ARS			*0.066*	*0.540*	***0.003***
Root biomass/Shoot biomass	R/S			*0.397*	*0.068*	*0.086*
Depth/Width of the root system	D/W			***0.027***	***0.029***	*0.062*
Depth/Width/Root biomass	DW/RB			*0.347*	***0.011***	*0.909*

Table S2. GLM (Generalized linear model) testing significant variations in the total length of fine roots, Number of fine roots, and total root length of *Q. ilex* seedlings during two months in presence of ectomycorrhizal symbiosis of *P. tinctorius* and conspecific litter at different stages of decomposition.

treatments	The total length of fine roots		N° of fine roots		Total root length	
	F	***p***	***F***	***p***	***F***	***p***
Intercept	**252.8456**	***0.000000***	**382.8198**	***0.000000***	**432.5799**	***0.000000***
ECM	**19.0983**	***0.000066***	**22.7757**	***0.000017***	**25.5213**	***0.000007***
Litter	0.0475	*0.953665*	1.7781	*0.179905*	0.5802	*0.563646*
week of growth	1.3507	*0.269108*	**9.4895**	***0.000050***	1.0156	*0.394054*
ECM*Litter	**13.5504**	***0.000022***	**18.0763**	***0.000001***	**9.1856**	***0.000419***
ECM*week of growth	1.4087	*0.251707*	1.2899	*0.288606*	0.6407	*0.592594*
Litter*week of growth	1.6025	*0.167024*	1.9464	*0.092323*	**2.7618**	***0.021824***
ECM*Litter*week of growth	0.5342	*0.779553*	1.2259	*0.309768*	0.9316	*0.481133*
